# Retraction: Genetic characterization of the highlander Tibetan population from Qinghai-Tibet Plateau revealed by X chromosomal STRs

**DOI:** 10.1371/journal.pone.0280666

**Published:** 2023-01-19

**Authors:** The PLOS ONE Editors

Following the publication of this article and Correction [1, 2], concerns were raised regarding ethics approval and consent.

PLOS received copies of ethics approval and consent documents in follow-up discussions. These documents were not sufficient to resolve concerns about the informed consent procedure, and about whether this work received a study-specific ethics approval from the relevant committee or review board.

In light of these issues, which question the compliance of this article with the PLOS Human Subjects Research policy, the *PLOS ONE* Editors retract this article.

XNL and AA did not agree with the retraction. SH, WSAQ, MAA, DSA, ASJ, SAMA, and FJ either did not respond directly or could not be reached.

S1 Fig [1] contained images of maps for which the source was not cited. The source was not clarified in discussions with the authors, and it is not known if the images are offered under a CC-BY license. Therefore, at the time of retraction, the article [1] was republished to remove S1 Fig.

The article’s Data Availability statement says, “The Data Availability Statement is under discussion and will be provided in a forthcoming update to this article.” [1] As the article is being retracted, the Data Availability statement will not be updated.
